# A multi-pathway hypothesis for human visual fear signaling

**DOI:** 10.3389/fnsys.2015.00101

**Published:** 2015-08-24

**Authors:** David N. Silverstein, Martin Ingvar

**Affiliations:** ^1^PDC Center for High Performance Computing and Department of Computational Biology, KTH Royal Institute of TechnologyStockholm, Sweden; ^2^Stockholm Brain Institute, Karolinska InstitutetSolna, Sweden; ^3^Department of Clinical Neuroscience, Karolinska InstitutetSolna, Sweden

**Keywords:** fear, emotion, visual perception, attention, amygdala, neural pathways

## Abstract

A hypothesis is proposed for five visual fear signaling pathways in humans, based on an analysis of anatomical connectivity from primate studies and human functional connectvity and tractography from brain imaging studies. Earlier work has identified possible subcortical and cortical fear pathways known as the “low road” and “high road,” which arrive at the amygdala independently. In addition to a subcortical pathway, we propose four cortical signaling pathways in humans along the visual ventral stream. All four of these traverse through the LGN to the visual cortex (VC) and branching off at the inferior temporal area, with one projection directly to the amygdala; another traversing the orbitofrontal cortex; and two others passing through the parietal and then prefrontal cortex, one excitatory pathway via the ventral-medial area and one regulatory pathway via the ventral-lateral area. These pathways have progressively longer propagation latencies and may have progressively evolved with brain development to take advantage of higher-level processing. Using the anatomical path lengths and latency estimates for each of these five pathways, predictions are made for the relative processing times at selective ROIs and arrival at the amygdala, based on the presentation of a fear-relevant visual stimulus. Partial verification of the temporal dynamics of this hypothesis might be accomplished using experimental MEG analysis. Possible experimental protocols are suggested.

## Introduction

Understanding the neurological basis of human emotional processing is a challenging problem, not only because observed neural correlates span cortical and subcortical structures, but also because experimental data may come from many different sources, including anatomical studies, functional magnetic resonance imaging (fMRI), diffusion tensor imaging (DTI), electroencephalography (EEG), magnetoencephalography (MEG), positron emission tomography (PET), and electrophysiology. Of emotional behaviors and the underlying emotional circuits (LeDoux, 2000), visual fear processing and the underlying signaling is an interesting system to investigate because it is well-studied across several animal models and produces relatively high contrasts in human imaging studies. Integrating data across experimental modalities can provide the constraints necessary for more detailed and computational models. This study was initiated to help determine these spatial and temporal constraints. Furthermore, a well-defined model of visual fear signaling can serve as a scaffolding to be extended into other emotional states. Additionally, progress on mapping of fear signaling pathways and recurrent loops could enable new diagnostic and therapeutic techniques.

Fear is considered by some a basic emotion (Ekman, 1992; Vytal and Hamann, 2010), which may have evolved over time at least partially in response to perceived threats from predators or dominating individuals in social orders (Öhman, 1986). Fear processing can encompass conscious awareness, conscious perception, pre-conscious perception, subliminal processing, or a combination of them. This investigation focused on visual fear signaling and perception, although fear processing through awareness can occur without this, for example though situational reasoning or while dreaming (Robert and Zadra, 2014). Conscious visual perception suggests that the subject can recognize and identify a particular presentation stimulus. Pre-conscious visual perception suggests that the subject has a relatively strong neural response to the presentation across the visual cortex, but either is not yet consciously aware of it, or will miss it due to the absence of attention (Dehaene et al., 2006). If a pre-conscious percept never becomes conscious during a presentation trial, then the perception was also unconscious or non-conscious. Should there be no conscious perception while activation in the visual cortex was relatively weaker, the perception would be subliminal, as well as unconscious or non-conscious. If there was no conscious recognition nor neural correlates for pre-conscious or subliminal perception, then there was no perception or awareness of the stimulus. Experimental paradigms such as backward masking and attentional blink explore the differences between subliminal or pre-conscious and conscious perception, with and without emotional salience. While the neural correlates of consciousness are much debated (Chica and Bartolomeo, 2012), in the case of humans it appears to at least require participation of particular cortical regions, including visual and parietal cortex for conscious visual perception, in addition to parieto-frontal activity (Dehaene et al., 2006).

Many cortical and subcortical brain areas are believed to be involved in visual fear processing. On the subcortical side, they include the lateral geniculate nuclei (LGN), pulvinar nucleus of the thalamus, superior colliculus (SC), amygdala, periaqueductal gray (PAG), locus coeruleus (LC), and hypothalamus. On the cortical side, they include the hippocampus, visual cortex (VC), parietal cortex, orbitofrontal cortex (OFC), lateral and medial prefrontal cortex (PFC), insula and anterior cingulate cortex (ACC). In this study, the emotion of fear is assumed to be highly correlated with amygdala activation. Thus, the visual signaling of fear is assumed to occur along direct and indirect neural pathways to the amygdala, which once activated, generates further responses. Evolution likely optimized the primate response time on perceived threats to be as fast as possible, to improve survivability. Because fear and threat appraisal has neural correlates and activations across the brain, the long-range signaling likely propagates along the white-matter fasciculi, because these myelinated fibers have the fastest propagation speeds.

Fear processing has been hypothesized to have parallel “low road” and “high road” pathways, from fear conditioning and lesion studies on rats (LeDoux, 1996). The low road is thought to be fastest and completely subcortical, while also unconscious (Öhman et al., 2007), although there is debate on this (Pessoa et al., 2006; Pessoa and Adolphs, 2010). The high road traverses sensory and higher cortices, and can encompass conscious perception. Regarding the low road, evidence exists from blindsight patients that perceptual signaling travels from the retina to the SC and back to the pulvinar nucleus, before arriving at the lateral nucleus of the amygdala (Morris et al., 1999, 2001; Pegna et al., 2005). Anatomical evidence from tract tracing and electron microscopy in the tree shew shows projections from the SC to the lateral amygdala via the dorsal pulvinar (Day-Brown et al., 2010). In humans, projections were found between the SC and amygdala via the pulvinar *in vivo*, using DTI (Tamietto et al., 2012). Along this pathway, the SC is capable of image detection of fear-relevant stimuli at low spatial frequencies (Vuilleumier et al., 2003). A recent study found that neurons in the macaque pulvinar can respond selectively to snakes in 55 ms (Van Le et al., 2013), which is likely too short for a cortical route. It has also been found that the amygdala can be activated with low latencies from a fear-relevant stimulus in about 40–120 ms (Luo et al., 2010), perhaps along the low road.

**Figure 1:**
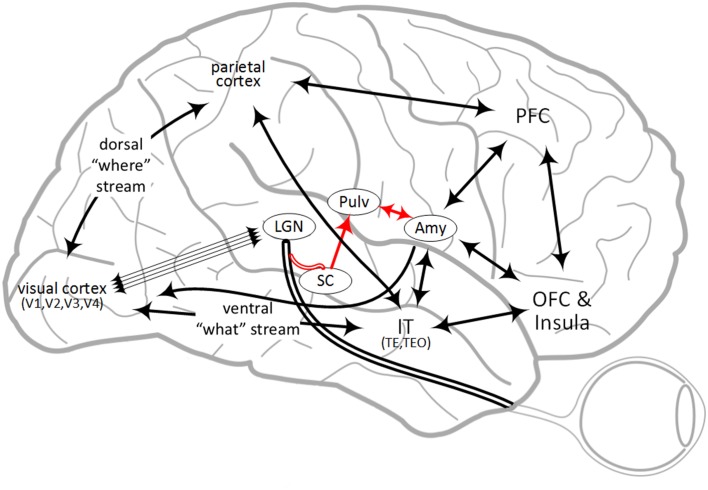
**Overview of some brain regions involved in fear processing and some of the connectivity between them**. Abbreviations are described in Table 1. The “low road” pathway is shown in red.

While dual pathways were initially observed in rats, there is functional evidence this applies to primates and specifically humans (Rudrauf et al., 2008; Garrido et al., 2012; Garvert et al., 2014) as well. Connectivity from anterograde and retrograde tracing studies in primates and diffusion tractography in humans appear to indicate multiple possible high-road pathways through multiple sensory cortices, as well as multi-area recurrence. An overview of some of this connectivity is illustrated in Figure 1. For vision in macaque monkeys, part of the visual stimulus travels from the LGN though the VC, along the ventral “what” pathway to the inferior temporal cortex (IT), where it directly projects to the amygdala (Webster et al., 1991; Baizer et al., 1993; Cheng et al., 1997; Ghashghaei and Barbas, 2002; Stefanacci and Amaral, 2002; Freese and Amaral, 2005). IT also projects to the OFC, parietal cortex and PFC (Webster et al., 1994). In addition, emotional pathways to the amygdala in macaque monkeys have been anatomically identified from the OFC and PFC (Carmichael and Price, 1995; Ghashghaei and Barbas, 2002; Barbas et al., 2003; Barbas, 2007; Cho et al., 2013). Thus, there is evidence for several different parallel pathways from IT to the amygdala, each of which may have different correlated behavioral characteristics and propagation times. Indeed, a “multiple-waves model” was proposed which reflects this connectivity between visual areas and areas involved in emotional processing (Pessoa and Adolphs, 2010). The present investigation seeks to further identify fear signaling pathways by first identifying functional evidence on relevant brain regions for fear, followed by identifying the evidence for connectivity between these regions which converge on the amygdala and then proposing information processing, signaling and latencies across this connectivity for behavioral differentiation. Finally, experiments are proposed to test and potentially modify this signaling hypothesis.

**Table 1:** **Abbreviations of anatomical primate brain regions and nuclei**.

Amygdala region	Amy	Amygdala
	Ce	Central nucleus
	M	Medial nucleus
	BLA	Basolateral complex
	LA	Lateral nucleus
	B	Basal nucleus
	AB	Accessory basal nucleus
	ITC	Intercalated cell masses
Basal ganglia	NAcc	Nucleus accumbens
	VSTR	Ventral striatum
Brainstem	LC	Locus coeruleus
Frontal lobe	ACC	Anterior cingulate cortex
	FEF	Frontal eye fields
	PFC	Prefrontal cortex
	lPFC	Lateral PFC
	mPFC	Medial PFC
	dlPFC	Dorsolateral PFC
	vlPFC	Ventrolateral PFC
	vmPFC	Ventromedial PFC
Inferior temporal lobe	IT	Inferior temporal cortex
	FFA	Fusiform face area
	TE	Anterior IT
	TEO	Posterior IT
Insular cortex	AI	Anterior insula
	Ia	Agranular field
	Id	Dysgranular field
	Ig	Granular field
Midbrain	PAG	Periaqueductal gray
	SC	Superior colliculus
	VTA	Ventral tegmental area
Occipital lobe	VC	Visual cortex
	V1	Primary visual cortex
	V2	Secondary visual cortex
	V3	Visual area V3
	V4	Visual area V4
	MT	Middle temporal area/V5
Orbitofrontal cortex	OFC	Orbitofrontal cortex
	lOFC	Lateral OFC
	mOFC	Medial OFC
	pOFC	Posterior OFC
Parietal cortex	LIP	Lateral intraparietal cortex
	IPL	Inferior parietal lobe
	IPS	Intraparietal sulcus
Thalamus	LGN	lateral geniculate nucleus
	Pulv	Pulvinar
	PI	Inferior pulvinar
	PL	Lateral pulvinar
	PM	Medial puvlinar

## Brain areas of fear processing

There is ongoing debate on what constitutes functional emotional processing in general in the primate and human brain. One approach considers basic emotions and the correlated locations of activity during these emotional behaviors (Phan et al., 2002; Murphy et al., 2003; Vytal and Hamann, 2010). Another approach investigates networks and groups of activity across a construction of emotional dimensions (Barrett and Wager, 2006; Kober et al., 2008; Lindquist et al., 2012). Still another view considers an evolutionary stratification into instinctual, learned and thought-related processes (Panksepp and Watt, 2011). The approach taken here considers the basic emotion of fear as activity across a distributed network of brain areas, but does not assume these areas are unique to fear. Further, this distributed activity may correspond with evolutionary stratification of instinctual, learned and thought-related signaling pathways, with progressively longer latencies. Of brain areas activated in functional imaging studies that include fear, those considered for visual signaling pathways are described.

The amygdala complex is conserved across vertebrates and particularly mammals (Moreno and Gonzalez, 2007; LeDoux, 2012). It appears to be necessary for fear processing in rats (LeDoux, 1996) and in primates, considering that damage may result in fearlessness, as was observed early in Rhesus monkeys with Klüver-Bucy syndrome, which entails bilateral lesions of the anterior temporal lobe [Klüver and Bucy, 1939, republished Klüver and Bucy (1997)]. A patient S.M. with bilateral amygdala lesions that occurred sometime after the age of 10, has had no fear experiences after this time while still exhibiting other emotions (Feinstein et al., 2011). Some studies of epileptic patients have shown that direct stimulation of the amygdala with electrodes can induce fear (Chapman et al., 1954; Lanteaume et al., 2007). While the amydgala can be activated during functional imaging experiments when a subject views happy faces (Killgore and Yurgelun-Todd, 2004), angry faces (Whalen et al., 2001; Pichon et al., 2008, 2009) and others, the effects of fearful faces are strongest (Whalen et al., 2001; Costafreda et al., 2008; Mattavelli et al., 2014). Functional meta-analysis of PET and fMRI emotional studies showed that fear-relevant stimuli specifically engaged the amygdala (Phan et al., 2002; Murphy et al., 2003). In addition, a study of nine human subjects with partial or complete bilateral amygdala damage showed significant impairment on recognizing fearful expressions, but not happy ones (Adolphs et al., 1999). However, at least one study of two human subjects did not find impaired recognition of fearful faces with complete bilateral amygdala lesions (Hamann and Adolphs, 1999). Still, recognizing fearful faces may not constitute experiencing fear.

The primate amygdala has both cortical and subcortical afferents and efferents and is composed of about 13 interconnected nuclei which can be divided into two major groups (Aggleton, 1985; Price et al., 1987; McDonald, 1998; Sah et al., 2003; Freese and Amaral, 2009). The evolutionarily older central (Ce) and medial (M) nuclei consist mostly of inhibitory medium spiny neurons (Martina et al., 1999), while the evolutionarily newer cortical-like nuclei are grouped as the basolateral complex (BLA) and a superficial nuclear group consist of pyramidal cells and interneurons (Hall, 1972). The nuclei in the BLA include the lateral (LA), basal (B), and accessory basal (AB) nuclei. The B and AB are also called the basolateral and basomedial nucleus, respectively. The BLA nuclei receive input and context from different cortical areas and the hippocampus, which appear to collectively compute possible danger and emotional salience, outputting via cortical efferents as well as through the Ce and M nuclei. The Ce projects to the hypothalamus (Ghashghaei and Barbas, 2002) and may activate it for release of stress hormones, as well as the PAG (Rizvi et al., 1991) for antinociceptive activity. Evidence exists in rats that severing connections between the BLA and Ce remove conditioned fear responses (Jimenez and Maren, 2009). Between some nuclei are the intercalated cell masses (ITC), which inhibit excitatory projections between the BLA and the Ce (Royer et al., 1999), lowering amygdala output activity. The amygdala has many reciprocal neocortical afferents and efferents and once activated, influences perception (Öhman et al., 2007). While IT projects to the amygdala, the amygdala projects back to the VC along the ventral stream from primary visual cortex (V1) to IT area TE (Carmichael and Price, 1995; Stefanacci and Amaral, 2002; Freese and Amaral, 2005, 2006) and can heighten visual awareness during activation, potentially assisting a pre-conscious fear-relevant stimulus in becoming consciously perceived (Vuilleumier and Driver, 2007). The B and AB nuclei also project to the ventral striatum (Cho et al., 2013).

The primate VC is composed of some 32 regions (Felleman and Van Essen, 1991), which have been grouped into a ventral “what” stream and a dorsal “where” stream (Ungerleider and Mishkin, 1982) or “what” and “how” streams (Goodale and Milner, 1992), all of which generally begin at V1. The focus of this investigation is on the ventral stream [V1↔(V2,V3)↔V4↔IT(TEO,TE)], where some aspects of fear-relevant stimuli can be pre-consciously perceived. In addition to serial and hierarchical processing of visual attributes, ventral visual areas also have complex connectivity, including substantial feedback projections and connections to and from subcortical areas. For example, V1 projects to V3, V4 and MT as well as V2 (Kravitz et al., 2013). Visual inputs from the retinas are delivered largely to V1 via the LGN, and propagate up to IT areas where object-level recognition can occur, and feedback from V1 to the LGN occurs as well (Briggs and Usrey, 2011). There is evidence the LGN also lightly projects directly to IT (Webster et al., 1993; Hernández-González et al., 1994) as well as V2, V4, and MT (Bullier and Kennedy, 1983; Sincich et al., 2004; Gattass et al., 2014). From IT, the ventral visual stream splits and propagates to several regions, including the parietal cortex (Distler et al., 1993; Webster et al., 1994), the LA and B nuclei of the amygdala (Webster et al., 1991; Baizer et al., 1993; Cheng et al., 1997; Ghashghaei and Barbas, 2002; Stefanacci and Amaral, 2002; Freese and Amaral, 2005), the lateral OFC (lOFC) (Webster et al., 1994; Kondo et al., 2003; Barbas, 2007) and the frontal eye fields (FEF) in the PFC (Webster et al., 1994; Schall et al., 1995). TE and to a lesser extent TEO also projects to vlPFC, although the function is not yet clear (Webster et al., 1994; Saleem et al., 2008; Gerbella et al., 2010) and may involve auditory processing (Medalla and Barbas, 2014).

The OFC is a highly interconnected region in the primate brain, with connections between sensory and prefrontal cortices as well as limbic structures, including bidirectional connections with the amygdala (Carmichael and Price, 1995; Rolls, 2005; Barbas, 2007; Price, 2007; Cho et al., 2013; Timbie and Barbas, 2014). While the amygdala learns about emotional and fearful stimuli, the OFC does this as well, while also computing a punishment or reward value (Rolls, 2004; Dolan, 2007; Rolls and Grabenhorst, 2008). The OFC is often divided into lateral/medial and anterior/posterior divisions. The lOFC receives visual stimuli from IT and other sensory areas, with the strongest visual projections from TE (Webster et al., 1994; Kondo et al., 2003; Barbas, 2007), likely over the uncinate fasciculus (UF). The lOFC is believed to be more active when processing aversive stimuli, while the medial OFC (mOFC) is more active when processing reward (O'Doherty et al., 2001; Kringelbach and Rolls, 2004). Learned punishments (or fears) and rewards are conditioned responses or secondary reinforcers from unconditioned stimuli or primary reinforcers (LeDoux, 1996; Rolls, 2005). The mOFC projects to the nucleus accumbens (NAcc) and likely stimulates it on expected reward. Along the anterior/posterior axis, simpler reinforcers are represented in the posterior area and become progressively more complex toward the anterior (Kringelbach, 2005). The posterior OFC (pOFC) has the strongest connections with the amygdala (Barbas, 2007; Barbas et al., 2011; Timbie and Barbas, 2014), with unidirectional projections from the pOFC to the ITC and bidirectional projections between the pOFC and the B and AB nuclei. The pOFC also receives inputs from sensory and olfactory cortices. While anatomical studies show strong pOFC connectivity, functional imaging studies typically have distinguished only between lOFC and mOFC (Kahnt et al., 2012), so it is assumed that pOFC and mOFC functional activity are blended together. Projections from other areas of the OFC also innervate the basal, AB, Ce, LA nuclei and ITC in the amygdala, while projections back to OFC originate in the basal, AB and LA nuclei.

The PFC is a large cortical area attributed to many functions in executive control. Along the medial-lateral axis, processing is self-referential to situational, along the ventral-dorsal axis, processing is emotional to cognitive and along the posterior-anterior axis, processing is more visceral to abstract. The PFC is typically divided into several regions, three of which will be focused on here. These are the medial PFC (mPFC), dorsolateral PFC (dlPFC) and the ventrolateral PFC (vlPFC). Activity in the mPFC has been correlated with self-referential processing (Gusnard et al., 2001) and extinction, while situational processing is more lateral (Ochsner et al., 2004). Lesions in the ventromedial PFC (vmPFC) have shown impairments in facial emotion recognition (Heberlein et al., 2008). Along the dorsal-ventral axis, the dlPFC has been implicated for abstract reasoning and working memory, while the vlPFC appears more focused on emotional regulation and self-control (Cohen et al., 2013). Internally, the mPFC, dlPFC, and vlPFC are all interconnected, and each also has varying connectivity to the OFC (Yeterian et al., 2011). The vlPFC in particular has strong connections to the lOFC, is adjacent to it, and sometimes considered overlapping (Petrides and Pandya, 2002; Cohen et al., 2013). The mPFC also has projections to the NAcc in the ventral striatum. In macaque, the mPFC has strong bidirectional connections to both the B and AB nuclei of the amygdala, while more laterally the PFC has only weak bidirectional connections with the B nucleus (Ghashghaei et al., 2007). Projections from the mPFC are excitatory, and there are two hypotheses on how they may also have an inhibitory effect on amygdala activity. The first suggests that since both the B and AB are cortical-like with pyramidal cells and interneurons, the interneurons may be directly targeted to inhibit the pyramidal cells disynaptically, while the second suggests that ITC cells could be targeted, inhibiting BLA to central nucleus projections (Quirk et al., 2003; Sotres-Bayon et al., 2004). The mPFC also targets the hypothalamus directly, with likely excitatory projections (Rempel-Clower and Barbas, 1998; Barbas et al., 2003).

The parietal cortex receives visual information from IT, with strong projections from TEO to the lateral intraparietal cortex (LIP) in the intraparietal sulcus (IPS) (Distler et al., 1993; Webster et al., 1994). The parietal cortex is critical for spatial awareness and damage is well known to cause hemineglect (Driver and Vuilleumier, 2001; Shinoura et al., 2009). The superior longitudinal fasciculus (SLF) connects the parietal and pre-frontal cortices bidirectionally with tracts I, II, and III (Makris et al., 2005; Kamali et al., 2014). Tract II may be most relevant for spatial awareness, passing visual information on to mPFC and working memory in the dlPFC, as well as more laterally to the vlPFC. Anatomically, the parietal cortex does not appear to project to the amygdala directly (Stefanacci and Amaral, 2002), but is part of other pathways to the amygdala. The posterior parietal does project to the medial temporal lobe, including the hippocampus (Kravitz et al., 2011), which is adjacent to the amygdala. To achieve conscious visual perception, it is likely that the visual signal needs to propagate via the parietal cortex through the frontoparietal network, which is thought to act as an attentional gate to the PFC (Kranczioch et al., 2005; Sergent et al., 2005; Dehaene and Changeux, 2011). However, it has not yet been fully demonstrated that the PFC is required for conscious perception. Evidence shows that the PFC is important for top-down attentional control, but responses to bottom-up stimuli have been observed with at least lateral PFC lesions (Rossi et al., 2009; Zanto et al., 2011).

The insula is important for interoceptive processing and has a diverse set of functions, including feeding, touch, vocalization and feelings of disgust. The anterior insula (AI) can be decomposed into the agranular ventral anterior (Ia), dysgranular dorsal anterior (Id) regions, while the posterior insula contains the granular posterior (Ig) region (Augustine, 1996; Deen et al., 2011). During human emotional experience and effortful tasks, the AI and ACC have shown conjoint activity in fMRI studies (Medford and Critchley, 2010; Gu et al., 2013; Engström et al., 2015). It has been hypothesized that primary interoceptive awareness such as pain and touch is represented in the posterior insula, while becoming more abstract along a posterior-anterior axis (Craig, 2009). In the macaque, the AI has strong projections to the LA, B and Ce of the amygdala (Stefanacci and Amaral, 2002), and also receives strong reciprocal input (Mufson et al., 1981; Amaral and Price, 1984). However, the AI is about 10–36% larger relative to brain mass in primates with larger brains such as humans and great apes, and some parts of the AI may be specialized (Bauernfeind et al., 2013). Evidence exists in macaque that among cortical areas, the insular projection from Id to Ce is unique (Stefanacci and Amaral, 2002). Ia has at least unidirectional projections to dlPFC and vlPFC (BA 47/12) while Id also has bidirectional connections with lOFC, pOFC, and PFC (Mesulam and Mufson, 1982; Flynn et al., 1999) and is functionally connected to the cognitive control network (Dosenbach et al., 2007). The AI projects to the ventromedial part of the ventral striatum, while the posterior insula projects to the dorsolateral striatum (Chikama et al., 1997). AI also receives input from the ventral medial (mediodorsal) nucleus of the thalamus (Flynn et al., 1999). In addition, the insula has moderate to heavy projections to the parietal lobe (Mesulam and Mufson, 1982; Flynn et al., 1999).

The pulvinar nuclei of the posterior thalamus consists of about 40% of the thalamus in humans and are typically split into inferior (PI), medial (PM), lateral (PL), and anterior (or oral) anatomical sections. For vision, the ventrolateral PL and central lateral PI have a retinotopic representation (Bender, 1981; Shipp, 2003; Li et al., 2013) and have dominant bidirectional connectivity with the ventral stream, whereas other parts of the PI are more interconnected with the dorsal stream (Shipp, 2003). Projections from the SC terminate mostly in PI, but in PL as well (Kaas and Lyon, 2007). The PL in particular can gate and control activity in V1 (Purushothaman et al., 2012). The dorsal medial PL is interconnected with the inferior parietal and PFC and the PM is interconnected with the cingulate, IT, OFC, PFC, insula and amygdala (Grieve et al., 2000; Kaas and Lyon, 2007). In the monkey thalamus, the PM alone projects to the B and LA nuclei of the amygdala (Jones and Burton, 1976; Romanski et al., 1997). Some have argued that the pulvinar may be involved in attention and awareness but not necessarily automaticity during emotional processing (Padmala et al., 2010; Pessoa and Adolphs, 2010), while others claim that while it replicates some cortical connectivity, its function is more regulatory in nature and can help sustain recurrent activity (Shipp, 2003) and cortical synchrony (Saalmann et al., 2012). This study focuses on the signaling pathways between activated neural correlates of fear responses, and less so on recurrent and sustained activity. While this signaling is not incompatible with recurrent activity through the thalamus and pulvinar, we do not explicitly represent the replicated cortical connectivity of the thalamus in the pathways, except for some connectivity within the visual stream.

## A multiple high road hypothesis

Among the many emotional pathways in the human brain, several appear to be utilized in fear signaling. These may not be unique to fear and may also be used in other emotional behaviors. In addition, some emotional pathways may inhibit others, while some might have mixed activity. For example, fear and anger have overlapping pathways (Pichon et al., 2009) and may coexist while fear and happiness are less likely to do so. Macaque anatomical and human tractography data indicate several different pathways from the visual sensory system to the amygdala, including the low and high roads. These are likely emotional pathways which might be utilized in fear processing. Functional connectivity from imaging studies can help validate this. However, even among fear pathways, activation may depend on the type of stimulus and response, and vary across different experimental protocols. In human imaging studies, pictures of fearful, emotional, and neural faces are often used (Ekman and Friesen, 1976; Fusar-Poli et al., 2009), although this may entail the perception of fear, and not necessarily the actual experience of fear. Some stimuli might be conditioned to produce a fear response while other unconditioned stimuli may produce a cognitive or situationally driven fear response. An ongoing challenge is to determine what stimulus and response corresponds to activation in what pathways.

**Figure 2:**
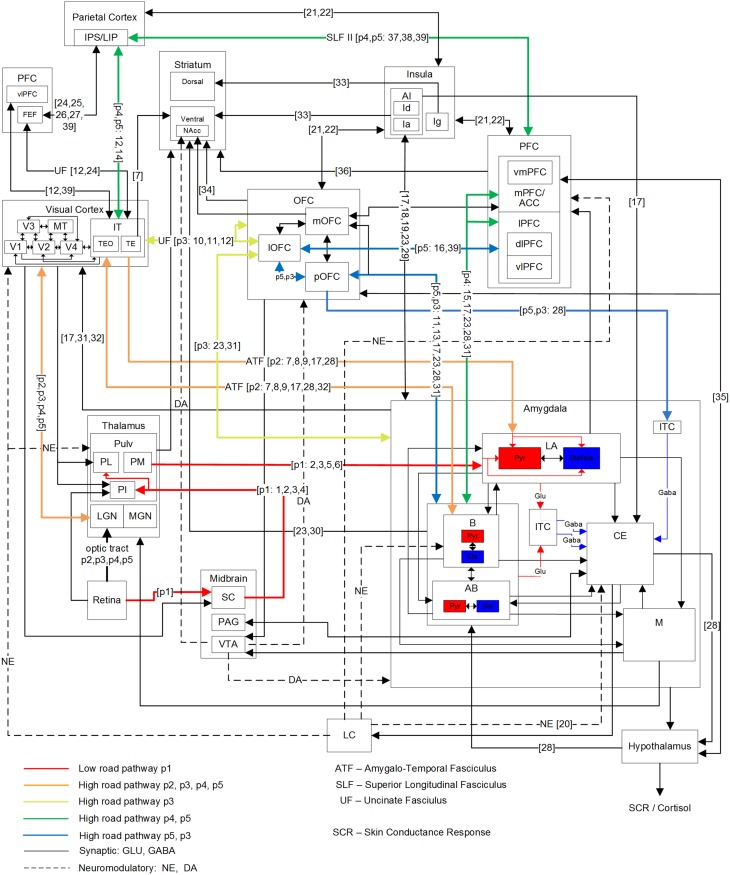
**Primate network diagram showing a subset of relevant brain areas and projections for visual fear signaling**. Anatomical abbreviations are described in Table 1. The proposed fear signaling pathways are labeled p1–p5. Projection preferences 1–39 are listed in Table 2, which are not exhaustive. Hippocampus and most ACC circuits are excluded. The amygdala superficial cortical nuclei are also excluded. For clarity, PFC and ITC groups appear more than once. Projections may connect a nested group when there are multiple internal sources/destinations or the source/destination is uncertain.

Figure 2 shows a detailed subset of known connectivity between brain regions involved in fear processing, as well as the hypothesized fear signaling pathways for vision, all of which exhibited experimental evidence for both functional and direct anatomical connectivity. However, in addition to the pathways illustrated in Figure 2, others likely exist. Connections with the adjacent hippocampus (Saunders et al., 1988) were not considered, which are known to play a role in fear conditioning, based on spatial cues and memory (LeDoux, 2000; Phelps and LeDoux, 2005; Alvarez et al., 2008). A fearful place for example, might trigger a visual signal via the posterior parietal to the hippocampus and on to the amygdala, but there is not yet functional evidence for this in primates. The FEF is bidirectionally connected to the VC, IT and parietal cortex, and projects to the PFC as well. In addition to saccade control, the FEF is known to modulate attention (Chica et al., 2014), but does not project to the amygdala directly. Hypothesized magnocellular projections to the mOFC have been suggested to preferably transfer low spatial frequency features before IT might see it (Bar et al., 2006; Kveraga et al., 2007; Chaumon et al., 2014), but the anatomical evidence is still uncertain. While IT and particularly TE strongly projects to OFC, weak projections were found from the IPS area of the posterior parietal to the lOFC in monkeys (Morecraft et al., 1992), along with projections to the FEF from the superior temporal sulcus (Schall et al., 1995). Still, in humans, the inferior fronto-occipital fasiculus (IFOF) appears to project from the parietal dorsal stream to the lateral and basal OFC (Martino et al., 2010; Sarubbo et al., 2013). A sub-cortical route to OFC is possible through the amygdala or pulvinar, although the amygdala is not likely to transfer details in low spatial frequency features. It is also possible for the signal to arrive at the mOFC rapidly via IT, which can be activated in 80–110 ms (Rolls et al., 2005), possibly along the inferior longitudinal fasciculus (ILF) or even directly via the LGN (Webster et al., 1993), followed by traversing the UF to the OFC. A pathway to the amygdala through the insula is also possible, since both the OFC and PFC project to it directly. Movement can induce fear as well. Visual-vestibular input can induce fear when falling, for example (Coelho and Balaban, 2015). There also exists evidence of a vestibular pathway to the amygdala. The vestibular nuclei have been found to project to the parabrachial nucleus in primates (Balaban et al., 2002), which in turn have direct bi-directional connections to the amygdala Ce, as well as indirect connections via the hypothalamus and OFC (Balaban and Thayer, 2001).

**Table 2:** **Numbered references for projections in Figure 2**.

1	at	Primate	Kaas and Lyon, 2007
2	at	*Tupaia belangeri*	Day-Brown et al., 2010
3	dti	Human	Tamietto et al., 2012
4	fp	Human	Morris et al., 1999
5	at	*Saimiri sciureusMacaca mulatta*	Jones and Burton, 1976
6	at	*Macaca mulatta*	Romanski et al., 1997
7	at	*Macaca fuscata*	Cheng et al., 1997
8	at	*Macaca mulatta*	Webster et al., 1991
9	at	*Macaca mulatta*	Baizer et al., 1993
10	at	*Macaca fascicularis*	Kondo et al., 2003
11	at	Primate	Barbas, 2007
12	at	*Macaca mulatta*	Webster et al., 1994
13	at	*Macaca mulatta*	Timbie and Barbas, 2014
14	at	*Macaca mulatta*	Distler et al., 1993
15	at	*Macaca mulatta*	Ghashghaei et al., 2007
16	fm	Human	Golkar et al., 2012
17	at	*Macaca mulatta*	Stefanacci and Amaral, 2002
18	at	*Macaca mulatta*	Mufson et al., 1981
19	at	*Macaca fascicularis*	Amaral and Price, 1984
20	at	Sprague-Dawley rat	Chen and Sara, 2007
21	at	*Macaca mulatta*	Flynn et al., 1999
22	at	*Macaca mulatta*	Mesulam and Mufson, 1982
23	at	*Macaca fascicularis*	Cho et al., 2013
24	at	*Macaca mulattaMacaca fascicularis*	Schall et al., 1995
25	at	*Otolemur garnetti*	Fang et al., 2005
26	at	*Macaca mulattaMacaca fascicularis*	Stanton et al., 1995
27	at	*Cebus apella*	Clower et al., 2001
28	at	*Macaca mulatta*	Ghashghaei and Barbas, 2002
29	at	*Macaca mulatta*	Höistad and Barbas, 2008
30	at	*Macaca*	Fudge et al., 2002
31	at	*Macaca*	Carmichael and Price, 1995
32	at	*Macaca fascicularis*	Freese and Amaral, 2005
33	at	*Macaca nemestrinaMacaca mulatta*	Chikama et al., 1997
34	at	*Macaca nemestrinaMacaca mulatta*	Haber et al., 1995
35		*Macaca mulatta*	Barbas et al., 2003
36	dti	Human	Leh et al., 2007
37	dti	Human	Makris et al., 2005
38	dti	Human	Kamali et al., 2014
39	at	*Macaca fascicularis* *Macaca nemestrina* *Macaca mulatta*	Gerbella et al., 2010

*at, anatomical tracing; dti, diffusion tensor imaging; fp, functional PET; fm, functional MRI*.

In addition to the low-road pathway described earlier (Figure 1 in red; Figure 2, pathway p1), four additional cortical high-road pathways (Figure 2, p2–5) for visual fear signaling were identified. All the cortical pathways include part of the ventral stream, traversing through the LGN to V1 and propagating up the visual hierarchy to IT. From IT, they branch off and traverse different brain areas before arriving at the amygdala. Elements of the dorsal stream were not included, except for LIP/IPS. The pathways were also confined to a single hemisphere for simplicity, not including cross-overs to the contralateral side. While the low road has evidence of being unconscious, some cortical pathways may be unconscious as well. As seen in hemineglect and attentional studies, conscious visual perception likely requires gating through the frontoparietal network (Dehaene et al., 2006).

The shortest cortical pathway (p2) projects from IT, directly to the LA and B nuclei of the amygdala, and is excitatory. There are two possible variations of this, because anatomical projections from IT to the amygdala exist through both TEO and TE. In the case of presented face stimulation, the fusiform face area (FFA) is proximal to TEO, so this is one possible route for emotional faces, and likely the most salient one. The next shortest cortical pathway (p3) projects from IT to OFC, and then to the amygdala. TE projects strongly to lOFC, in addition to pOFC and mOFC. TEO projects to lOFC as well. While lOFC projects to the amygdala directly, an additional possible route is traversing through the pOFC to both the BLA and ITC masses in the amygdala. This can enable or inhibit amygdala activation generally, depending on conditioning and any extinction (Sotres-Bayon et al., 2004; Li et al., 2009). Both the p2 and p3 pathways are postulated to encompass pre-conscious perception, though they may engender a feeling when activated. Damage to the p2 and p3 pathways are hypothesized to cause Capgras syndrome, where patients lose the emotional response to seeing family members, and think they are impostors (Young et al., 1993; Hirstein and Ramachandran, 1997). Previous experiments disrupting IT processing suggest p2 and p3 pathways are also disrupted. In a binocular rivalry experiment, a suppressed image was either a fearful face or chair. When contrasted, the fearful face showed activation along the low road, but no differential activity in the FFA (Pasley et al., 2004). In a subsequent experiment where conscious perception was suppressed with a combination of binocular rivalry and motion flash suppression while contrasting fearful faces and houses, differential activity was seen in the VC and FFA, along with the FEF, IPL, and insula (Troiani et al., 2014). These experiments show neural correlates corresponding to pathway p2, assuming the observed insula activation was via an amygdala efferent. The other two hypothesized signaling pathways to the amygdala both project from IT to LIP in the parietal cortex and through the frontoparietal gateway to the PFC. The mPFC is known to be bidirectionally connected to the amygdala BLA, with stronger unidirectional connections from the ACC to BLA (Ghashghaei et al., 2007).

Signaling pathway p4 innervates the mPFC on route to the amygdala and is believed to be partially responsible for fear learning and extinction (Phelps et al., 2004). However, the mPFC may receive a fear-relevant visual stimulus stream from IT via both the frontoparietal network and through the OFC (branching from pathway p3), which can propagate up an expected aversive or appetitive value. If visual awareness from the frontoparietal stream does not meet expectations (i.e., a threat is not real), then to achieve extinction, the mPFC may down-regulate the amygdala and signal the OFC to reduce the encoded value. Amygdala BLA to mPFC signaling may also up-regulate attention when a fear-relevant stimulus is present. The anticipation of an unpredictable and unlearned pain stimulus has been found to increase activity in the ACC, vmPFC and PAG, while an expected learned pain stimulus showed reduced ACC and vmPFC activity (Hsieh et al., 1999). Some disorders such as post-traumatic stress disorder (PTSD) can show resistance to extinction and lower activity levels in the mPFC during extinction recall (Koenigs and Grafman, 2009; Milad et al., 2009). vmPFC lesions have also been implicated in mood and anxiety disorders, where patients exhibited higher amygdala responses to adverse images. This indicates that the vmPFC may be important for some fear regulation, particularly, with regards to self (Heatherton, 2011; Motzkin et al., 2014).

The final pathway (p5) enables inhibitory cortical control of fear from fear-relevant visual stimuli. From the frontoparietal network, it traverses the dlPFC to the vlPFC, then projecting down to the lOFC and through the pOFC to ITC masses of the amygdala, for inhibition. This pathway might also provide excitatory cortical control of fear via projections from pOFC to the B and AB nuclei, but there is not yet experimental evidence for this. Higher vlPFC and lOFC activity are correlated with emotional control (Phan et al., 2005; Hooker and Knight, 2006; Cohen et al., 2013) and damage to these areas can result in a loss of inhibitory emotional control. The vlPFC has been found to be negatively correlated with activation of the amygdala and mOFC during reappraisal (Ochsner et al., 2002). Activity in the lOFC has also been correlated with reappraisal of negative stimuli, while activity in the dlPFC was active more generally during reappraisal (Golkar et al., 2012). Emotional regulation has been found to be both willful and automatic, and data shows that willful regulation has higher activity in lateral PFC (lPFC) and automatic regulation has higher activity in the mPFC (Etkin and Wager, 2007; Phillips et al., 2008). Dysregulation may also occur with this pathway. For example, it was found that phobic subjects showed hypoactivity in the dlPFC and lOFC (Carlsson et al., 2004) as well as mPFC (Hermann et al., 2007, 2009). However, in addition to activation of the vlPFC and down-regulation of the amygdala during some effortful regulation tasks with negative emotional stimuli, some studies have found possible mediation via the vmPFC (Urry et al., 2006; Johnstone et al., 2007). While this may be similar to pathway p4, it does not explain lOFC activity in some studies (Phillips et al., 2003; Carlsson et al., 2004; Wager et al., 2008; Golkar et al., 2012).

**Figure 3:**
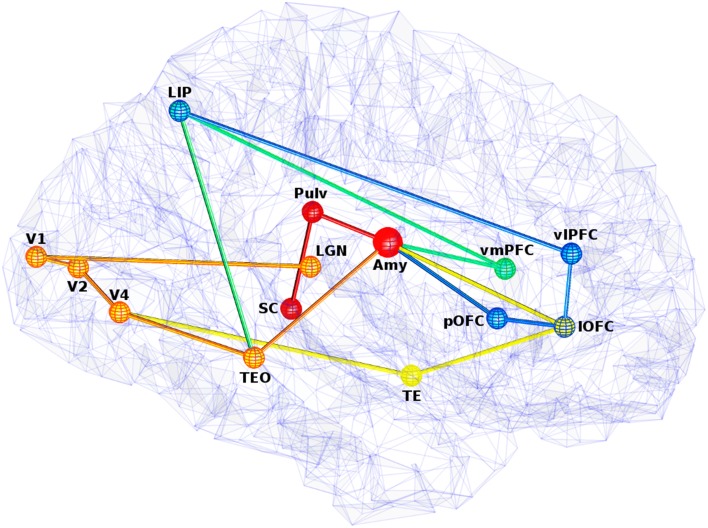
**View of five visual fear signaling pathways to the amygdala**. Abbreviations are described in Table 1. Pathways p1 is red, p2 is orange, p3 is orange to yellow at V4, p4 is orange to green at TEO, and p5 is orange to green at TEO, then blue at LIP. Projections between ROIs are shown as straight for simplicity, but these represented white matter fibers are typically curved with slightly longer lengths. Some ROI positions were slightly altered for clarity.

All five of these pathways as illustrated in Figure 3 have different lengths and latencies, so fear-relevant signals will arrive at the amygdala at different times. As this is happening, projections away from the amygdala will also have a behavioral effect, releasing norepinephrine via the LC for alertness, upregulating attention and boosting activity in recurrent loops. Amygdala efferents to PFC can also initiate reappraisal, which can either reduce or increase the fear response, depending on the perceived danger.

## Predicted fear signaling pathway latencies

Given the hypothesized fear signaling pathways, signal propagation latencies between regions of interest (ROI) along these pathways were predicted, as well as the total arrival times at the amygdala. To achieve this, coordinates of anatomical structures relevant to the pathways were represented with selective ROIs from the brain imaging literature. These ROIs are listed in Table 3. However, coordinates for these anatomical regions can vary considerably from study to study, depending on individual differences, experimental contrasts and analysis techniques. Thus, they are meant as approximations. ROIs along the pathways are connected with either fiber tract fasciculi or long-range projections. These projections are generally curved in and around anatomical areas and are not typically completely straight. While DTI techniques render and trace some of these fibers, the curvature lengths between ROIs have yet to be determined. Thus, as a first approximation of fiber tract lengths between ROIs, Euclidian distance was used, although the actual lengths would typically be at least slightly longer. Length estimates between ROIs can be seen in Table 4.

**Table 3:** **Regions of interest in visual fear processing (in Talairach space)**.

**Region**	**ROIs (right)**	***x***	***y***	***z***	**Contrast**	**References**
	Amygdala	25	−7	−10	Fear conditioning	Milad et al., 2007
	VSTR	14	8	−2	Amygdala anti-correlated	Satterthwaite et al., 2011
	PAG	0	−32	−10	Phobic vs. fear	Carlsson et al., 2004
	LC	9	−42	−30	Hungry vs. satiated	Mohanty et al., 2008
Vision	LGN	22	−25	−1		Hess et al., 2009
	Pulvinar	19	−24	14	Discrimination	Fischer and Whitney, 2009
	SC^*^	12	−28	−14	Fearful vs. neutral eyes	Morris et al., 2002
	V1	7	−88	0	Attention	Martínez et al., 2001
	V2	7	−78	−3	Attention	Martínez et al., 2001
	V4	19	−70	−11	Attention	Martínez et al., 2001
	FFA (TEO)	38	−43	−18	Faces vs. places and obj.	Mur et al., 2010
	aIT (TE)	35	−3	−25	Faces vs. baseline	Mur et al., 2010
OFC	pOFC	38	19	−11		Kober et al., 2008
	lOFC	51	31	−10	Maintain vs. suppress	Phan et al., 2005
	mOFC	9	33	−12	Attractive vs. unattractive	Bray and O'Doherty, 2007
PFC	vmPFC	8	15	−12.5	Fear conditioning	Milad et al., 2007
	dlPFC	40	31	20	Awareness vs. non-aware	Sahraie et al., 1997
	vlPFC	55	32	9	Pop-out perception	Eriksson et al., 2004
Other	IPL	52	−25	27	What vs. where	Harrison et al., 2010
	LIP	31	−71	45		Simpson et al., 2011
	ACC	4	28	16	Phobic vs. fear	Carlsson et al., 2004
	Insula	33	−23	−2	Disgust and fear cond.	Klucken et al., 2012

* *Converted from Montreal Neurological Institute (MNI) coordinates. Abbreviations are described in Table 1*.

**Table 4:** **Estimated distances along each visual fear signaling pathway edge, between ROI nodes**.

**Pathway afferents to amygdala**	**ROI (src)**	**ROI (dst)**	**Estimated length (mm)**
p1 sub-cortical	SC	Pulvinar	28
	Pulvinar	Amygdala	30
p2 IT	LGN	V1	65
	V1	V2	10
	V2	V4	16
	V4	FFA (TEO)	34
	FFA (TEO)	Amygdala	39
p3 OFC	V4	aIT (TE)	70
	aIT (TE)	lOFC	51
	lOFC	Amygdala	54
p4 PFC EX	FFA (TEO)	LIP	69
	LIP	vmPFC	106
	vmPFC	Amygdala	28
p5 PFC REG	FFA (TEO)	LIP	69
	LIP	vlPFC	112
	vlPFC	lOFC	19
	lOFC	pOFC	18
	pOFC	Amygdala	29
**AMYGDALA EFFERENTS**			
	Amygdala	LC	43
		V1	84
		V4	63
		FFA (TEO)	39
		vmPFC	28
		pOFC	29
		Insula	20
**OTHER SEGMENTS**			
	TE	vlPFC	52

*Also includes the estimated lengths of some amygdala efferents. Abbreviations are described in Table 1*.

**Figure 4:**
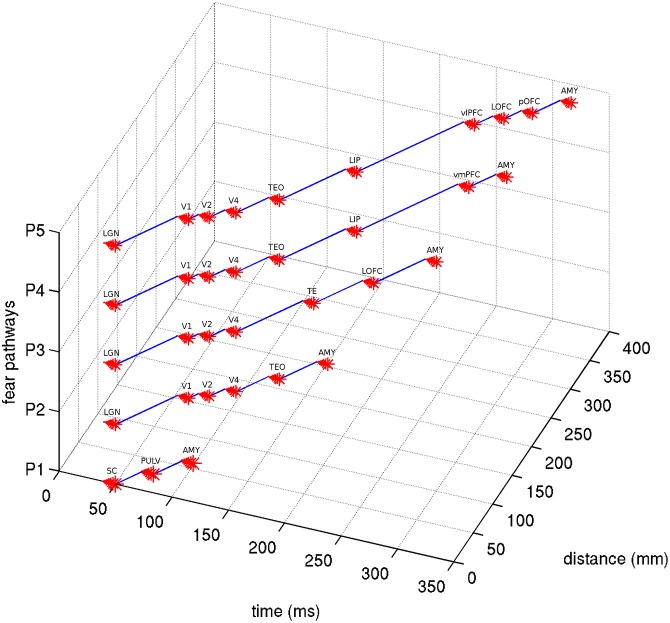
**Latency estimates of the five visual fear signaling pathways**. Assumes a latency of 40 ms from retina to LGN/SC, synaptic integration time of 10 ms at each ROI node and a propagation speed of 2 m/s. Abbreviations are described in Table 1.

Given a length estimate of each pathway edge, the total length of each pathway can be estimated, starting from the LGN or SC and ending at the amygdala. With simplifying assumptions that the neural propagation speed along the fasciculi is relatively uniform, and the synaptic integration time at each ROI is similar, a rough estimate can be made for the total latency of each fear pathway. Evidence exists that neural signaling propagation speeds in human cortical fibers may be around 2 m/s (Reed et al., 2004), although this is uncertain. In macaques, feedforward and feedback conduction velocities between areas V1 and V2 have been found to be about 3.5 m/s (Girard et al., 2001). Neural integration time has previously been assumed to be about 5–10 ms within areas of primate VC (Nowak and Bullier, 1997). 10 ms was used here, although cortical and subcortical neurons may behave differently in this respect. The signal propagation time of visual stimuli from retina to LGN has been found to be about 40 ms in humans (Krolak-Salmon et al., 2003) and comparable to macaques, which have been measured faster at 33 ms (Lamme and Roelfsema, 2000). Adding this latency to the estimated pathway latencies can predict actual latencies for signal arrival at the amygdala, which can be seen in Table 5. By using these assumptions for neural propagation speed and integration time, it is possible to estimate the temporal signal progression of each of the pathways, as can be seen in Figure 4.

**Table 5:** **Predicted pathway latencies for arrival at the amygdala, where *e* is the latency between the retina and LGN/SC, *s* is the neural propagation speed in m/s and *i* is the synaptic integration time at each ROI node**.

**Fear pathway**	**Nodes**	**Estimated length (mm)**	**Estimated latency**	**Total latency (ms) *e* = 40 ms; *s* = 2 m/s; *i* = 10 ms**	**Total latency (ms) *e* = 40 ms; *s* = 3 m/s; *i* = 10 ms**	**Total latency (ms) *e* = 40 ms; *s* = 3 m/s; *i* = 15 ms**
p1	3	58	*e* + 58/*s* + 2*i*	89	79	89
p2	6	164	*e* + 164/*s* + 5*i*	172	145	170
p3	7	266	*e* + 266/*s* + 6*i*	233	189	219
p4	8	328	*e* + 328/*s* + 7*i*	274	219	254
p5	10	372	*e* + 372/*s* + 9*i*	316	254	299

## Discussion

We have put forward a hypothesis of a multiple pathway model for fear processing and suggest that this multiplicity has evolved as part of the evolutionary drive to process and regulate fear reactions in a sophisticated manner and thereby move away from the reflex automata. To test whether these hypothesized fear signaling pathways exist or not, experimental protocols might use human imaging techniques such as MEG and fMRI. Further, if the same experimental protocols were used on both in the same analysis, fMRI could better localize the ROIs on specific behavioral contrasts and MEG could measure the temporal dynamics. Even better would be if the same subjects could be used, taking into account any individual differences and testing effects.

Protocols can be designed to selectively test for activation or deactivation of these signaling pathways. While different protocols might activate one or several of these parallel pathways simultaneously, an interesting finding would be if particular protocols show higher activity in particular pathways. To selectively test activation of the low road (pathway p1) or p2 and p3 pathways, backward masking might be employed for comparing activity between conscious and subliminal (or unconscious) perception of fear-relevant and neutral stimuli, as in Carlsson et al. (2004). This can be done by contrasting exposure times using a stimulus onset asynchrony (SOA) of near 20 ms with a longer one such as 100 ms. The category and features of the fear-relevant stimuli may determine which pathways are activated during backward masking. For example, eyes, spiders and snakes might activate pathway p1 whereas fearful faces might activate pathway p2 and p4 and more complex conditioned images might activate pathway p2 and p3. These activations may be distinguishable by signal arrival time at the amygdala, as well as contrasted activity.

For example, one study found a peak of gamma oscillations in the amygdala 135 ms after presentation of fearful vs. neutral faces and significant activity at 105 ms (Sato et al., 2011). Such latencies may indicate pathway p1 or p2 activation, because the other signaling pathways have longer estimated propagation times. Attentional gating is believed to occur later in the visual processing stream, after IT activation but between the parietal cortex and PFC. Attentional blink can happen when two expected visual targets, T1 followed by T2, are presented between 200 and 500 ms apart. Emotionally salient targets have been shown to modulate the attentional blink (Trippe et al., 2007; Ciesielski et al., 2010; Schwabe et al., 2011; Qian et al., 2012). An emotionally salient T1 has been found to increase the blink while an emotionally salient T2 has been shown to reduce it (Schwabe et al., 2011). In addition, a electrophysiology study showed a P300 event-related potential (ERP) was present when detecting T2 and was stronger when T2 was fear-relevant (Trippe et al., 2007). These studies seem to indicate that emotional stimuli are more likely to grab attention. Another ERP study showed that when contrasting a seen—absent T2, the ventral VC is fully active at 96 ms (P1 ERP), the parietal lobe is active at 180 ms (N1 ERP) and the PFC is active at 276 ms (N2 ERP) (Sergent et al., 2005). To contrast attentional pathway activations via pathway p4, recognition and non-recognition of T2 can be contrasted when T2 is fear-relevant and not. If the pathway hypothesis is correct, recognition of the fear-relevant target should show higher activity in pathway p4 than a neutral target would. Backward masking of a fear-relevant T2 may show higher activity in IT when the SOA is high enough for T2 to be perceived. Contrasting this with seen vs. unseen may also show differential activity along pathway p2 or p3 when comparing a masked fear-relevant T2 with an unmasked but missed (unseen) T2. However, higher neocortical activations might also occur from arousal and norepinephrine release via projections from the LC, which were activated earlier by lower subcortical pathways.

Other ways to separate activation of these pathways include using protocols for fear conditioning on the OFC pathway (pathway p3), reasoned fear (pathway p4), or fear regulation and suppression (pathway p5). Reasoned fear might occur when an unconditioned stimulus presents a situation where danger or a threat might occur and is recognized as such from visuospatial reasoning. Fear regulation and suppression may occur when the subject is told to actively try to suppress fear. Voluntary suppression of negative affect in an fMRI study showed higher activity in the PFC including the lateral areas and attenuated activity in limbic areas including the amygdala (Phan et al., 2005). The absence of regulation can also be contrasted between phobics and controls when presented with fear-relevant phobic stimuli.

Possible experimental protocols for testing these pathways are described in the Appendix. fMRI can provide spatial localization of ROIs and MEG can provide temporal and causal information. Ideally, if protocols for backward masking, attentional blink, fear conditioning, reasoned fear, and fear regulation could be performed on the same subjects, it may be easier to discriminate between different pathway activities. While it is unlikely that MEG can see signal propagation along axonal fibers, it can identify activity in ROIs along a pathway. However, ROIs and dipoles detected with MEG cannot be resolved if closer than 1 cm apart.

There is ongoing debate on whether MEG dipoles can be detected at the amygdala or not, but some experimental evidence exists (Cornwell et al., 2008; Luo et al., 2010; Balderston et al., 2013; Dumas et al., 2013). While the evolutionarily older Ce and M nuclei are composed of inhibitory neural populations, the newer nuclei of the BLA consist of pyramidal and stellate cells that may produce dipoles, although perhaps too deep and weak to be reliably detected. However, a recent MEG study found enhanced amygdala activity at 130–170 ms and later at 310–350 ms after visual presentation when contrasting the response to fearful and neutral faces (Dumas et al., 2013). Another MEG study of human responses to fearful and neutral faces found an amygdala response 40–140 ms unaffected by attentional load while a response 280–410 ms was modulated by attentional load (Luo et al., 2010). Intracranial EEGs are another technique which has shown some success in measuring amygdala activity. One intracranial ERP study found higher amygdala gamma-band activity at 50–150 ms, peaking at 135 ms when comparing the response to fearful and neutral facial expressions (Sato et al., 2011), while another intracranial study of emotional faces showed a fear response in the amygdala after 200 ms (Krolak-Salmon et al., 2004). These responses may be due to selective activations of the proposed pathways. Fearful facial expressions may activate pre-consciously along pathway p2 and perhaps the sub-cortical pathway p1 on some occasions, perhaps from the eyes. The later peaks may be the result of signal propagation across the SLF (frontoparietal gateway) and may show activation of pathways p4 and p5. While the predicted latencies for pathways p4 and p5 is a little shorter than what was observed for attended fear responses, this may be due to actual longer fiber lengths in the SLF, longer neural integration time in the PFC and signal switching to the contralateral side.

If the approximate timing of the separate signal arrivals at the amygdala can be predicted, they may be easier to find in temporal space. If it is not possible to detect the signal arrival at the amygdala, it could be approximated as a hidden node, with activation of the central nucleus validated by the skin conductance response (SCR) or other correlates. However, the SCR response is too slow to validate timing. Afferent fear signals that arrive at the amygdala first will propagate out through efferents such as the LC or VC, which may interfere with resolving other afferents. In addition, other pathways may have loops and recurrence, such as through the OFC. However, if the latencies between ROIs are resolvable, these effects could be isolated. On longer timescales, reappraisals can occur, producing even more signaling. Another possible experimental technique is to use transcranial magnetic stimulation to knock out ROI nodes along a signaling pathway, to see if the downstream signal propagation was interrupted.

### Conflict of interest statement

The authors declare that the research was conducted in the absence of any commercial or financial relationships that could be construed as a potential conflict of interest.

## Figures and Tables

**Figure A1:**
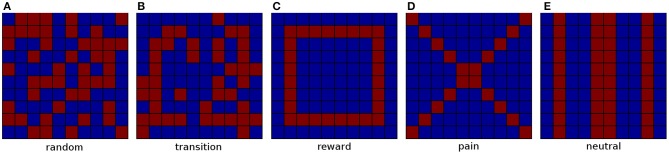
**Protocol for fear conditioning**. Starting from **(A)**, random transitions of pixels occur as in **(B)** once a second until an icon such as **(C,D)** or **(E)** fully forms. Icons are randomized for reward, pain (shock/thumb press), and neutral.

**Figure A2:**
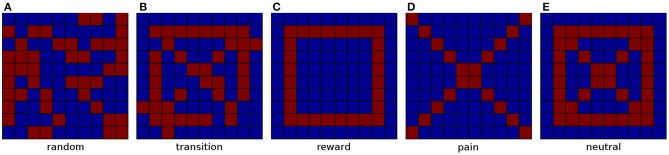
**Protocol for reasoned fear**. Starting from **(A)**, random transitions of pixels as in **(B)** occur once a second until an icon fully forms. Reward **(C)** and pain **(D)** icons are pre-conditioned, but neutral **(E)** is not. Transitions progress toward the neutral composite icon pattern **(E)**, before completing as **(E)**, or diverging to one of the pre-conditioned icons **(C** or **D**).

**Figure A3:**
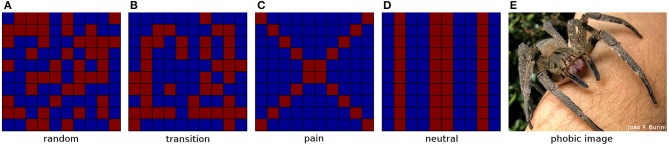
**Protocol for fear regulation**. Starting from **(A)**, random transitions of pixels occur as in **(B)** until an icon such as **(C)** or **(D)** fully forms or a phobic image such as **(E)** appears. Pain icons are pre-conditioned, but neutral icons or phobic images are not. Transitions progress toward the painful or neutral icon patterns and complete while delivering any painful stimulus randomly.

## Appendix

### Proposed multi-pathway experiments

The purpose of these proposed experiments is to explore signaling, cortical processing, and regulation of visually induced human fear and to determine if this processing corresponds to the proposed fear signaling pathways. It can encompass five different experiments which may validate these individual signaling pathways or combinations of them. However, if the experiments for each of these pathways can be performed on the same subjects, it may be easier in the analysis to discriminate between the different pathway activations. Combining experiments could also reduce the amount of any needed pain stimulation. While these experiments could be performed on MEG alone, duplicating the experiments on fMRI would provide higher spatial resolution as well. If the fear signaling pathways prove to be false, these data would provide enough spatial and temporal constraints to suggest alternatives. For example, if fear signaling is significantly gated through the pulvinar (Pessoa and Adolphs, 2010), or along the dorsal visual stream (Chaumon et al., 2014), differing signaling latencies with functional activity may provide a signature for discrimination.

Experiments are proposed for backward masking, fear-relevant attentional blink, fear conditioning, reasoned fear and fear regulation, each of which should show differential activity. For example, it is hypothesized that responses to a conditioned fear stimulus will show higher activation of a pathway from IT via the OFC to the amygdala and responses to reasoning-based fear will show higher activation of a pathway from IT via the parietial cortex, PFC and OFC to the amygdala. Because the reasoning-based pathway is longer, activation of the amygdala should have a longer latency than for conditioned fear responses. Parallel activation of signaling pathways may produce multiple pulses of amygdala activation at different time points. The SCR could be measured on all experiments to provide additional validation of amygdala activation.

### Experiment 1—fear conditioning

Fear conditioning experiments are often done using emotional and neutral faces. However, this may make it difficult to mix differently conditioned stimuli in subsequent protocols. Instead of faces, the subject can be presented with a 10 × 10 matrix of colored pixels which are set at random between trials (Figure A1A). Every second (for signal detection in fMRI), the color will be flipped on some pixels (Figure A1B) for transitioning until a recognizable pattern forms (Figures A1C–E). Some completed patterns will be associated with pain, others with reward and the remaining will not have salient associations. It is assumed that the anticipation of pain will usually generate some fear response. Pain can be inflicted with a thumb press, electric shock, or either at random. Uncertainty of the pain source may provide a better fear response. A reward might be a picture of a bank note that the subject can keep, or some percentage of it. Reward stimuli may provide a stronger contrast to fear (anticipated pain) than just the absence of pain would, because there may be competitive neural circuits. The SCR can be measured to determine if the pain and reward stimulus has been conditioned. It is hypothesized that a conditioned fear response from the icons would activate pathway p3. The response to icons can also be contrasted with emotional or conditioned faces, which may show higher activation through pathway p2.

### Experiment 2—backward masking

The low road (pathway p1) has been previously hypothesized to recognize simpler fear-relevant visual stimuli such as eyes, spiders and snakes via the pulvinar and SC. The FFA has been previously found to recognize facial features and may encompass pathway p2. Complex images may be recognized at TE as well as TEO/FFA and may encompass pathway p3. A backward masking protocol can investigate any differences in activations of these pathways with fear-relevant simple images (eyes, spiders, snakes), emotional faces and more complex conditioned images, when contrasting an SOA of around 20 ms with 100 ms. The fear conditioned icons from the previous experiment can be used if the experiments are done together. If amygdala activity can be detected with MEG, with intracranial EEG or with electrodes, any differences in latency alone may provide differentiation between pathways.

### Experiment 3—fear-relevant attentional blink

Contrasting fear-relevant target detection with non-detection in an attentional blink paradigm can help indicate where and when attentional gating occurs along pathway p4. Given a rapid serial visual presentation (RSVP) stream of numbers and targets every 100 ms (or lag), targets can be randomly selected to be neutral or fear-relevant. The subject must know a target to expect and indicate after a trial what was seen, with a button press or otherwise. Different categories of targets can be used, such as fearful and neutral faces or fear-conditioned and neutral icons from the fear conditioning experiment described earlier. It is expected that pathways p2 (for faces) or p3 (for icons) would activate on fear-relevant targets regardless of if they were consciously seen or not, and would typically generate an amygdala response. However, a second amygdala response should occur via p4 if a fear-relevant target is seen, with the longer predicted latency. Thus, contrasting neutral unseen targets with fear-relevant unseen targets might show pathway p2 or p3 activation and contrasting neutral seen with fear-relevant seen targets might show additional pathway p4 activation.

### Experiment 4—reasoned fear

For the reasoned fear protocol (Figure A2), pairs of pain-associated and reward-associated patterns from the fear conditioning protocol can be combined into composites. These composites can emerge from an initial random pattern and either complete as the full composite or transition to the pain or reward associated pattern. At some point, the subject should recognize the pattern shift through visuospatial analysis, which should evoke a response. In the first trial set, the subject can only observe the transitions, which can lead to reward, pain or no extra stimulation. In the second trial set, if the subject decides that the pain-associated pattern is emerging, the subject may press a button to stop the expected pain. But, if the subject is wrong, the pain will be administered regardless. This prevents just pushing the button to avoid the pain. The timing of the button press also helps mark the decision point in time, after a motor delay. At the realization of expected pain and near the decision point of pressing the button to avoid pain, pathway p4 should activate. Contrasting the first and second trial set may indicate activity from intended motor action.

### Experiment 5—fear regulation

The protocol for fear regulation (Figure A3) is similar to reasoned fear. The subjects are again presented randomly with the fear-conditioned or neutral stimuli, but without a button press. The subjects are told that if they can suppress their fear or anxiety, painful stimuli will not occur when pain-associated patterns are presented. They will be told that this can be determined by the SCR measurements. However, whether pain is administered or not will actually be determined randomly, in the case of multiple trials. The suppression of fear should show higher activation along pathway p5. If some phobic subjects are used, phobic images can also be presented randomly along with the pain and neutral stimuli. The response to phobic images can be contrasted with the neutral and fear-relevant stimuli. It is expected that active fear suppression would have stronger contrasts between fear-relevant and neutral stimuli as compared with phobic dysregulation.
